# Intestinal Dysbiosis and Rheumatoid Arthritis: A Link between Gut Microbiota and the Pathogenesis of Rheumatoid Arthritis

**DOI:** 10.1155/2017/4835189

**Published:** 2017-08-30

**Authors:** Gabriel Horta-Baas, María del Socorro Romero-Figueroa, Alvaro José Montiel-Jarquín, María Luisa Pizano-Zárate, Jaime García-Mena, Ninfa Ramírez-Durán

**Affiliations:** ^1^Servicio de Reumatología, Hospital General Regional 220, Instituto Mexicano del Seguro Social, Toluca, MEX, Mexico; ^2^Coordinación de Investigación en Salud, Delegación Estado de México Poniente, Instituto Mexicano del Seguro Social, Toluca, MEX, Mexico; ^3^Jefatura de División de Investigación en Salud, Unidad Médica de Alta Especialidad Hospital de Traumatología y Ortopedia, Instituto Mexicano del Seguro Social, Puebla, PUE, Mexico; ^4^Departamento de Nutrición y Bioprogramación, Instituto Nacional de Perinatología, Secretaría de Salud, Ciudad de México, Mexico; ^5^Departamento de Genética y Biología Molecular, Cinvestav, Av IPN 2508 Col Zacatenco, Ciudad de México, Mexico; ^6^Laboratory of Medical and Environmental Microbiology, Department of Medicine, Autonomous University of the State of Mexico, Toluca, MEX, Mexico

## Abstract

Characterization and understanding of gut microbiota has recently increased representing a wide research field, especially in autoimmune diseases. Gut microbiota is the major source of microbes which might exert beneficial as well as pathogenic effects on human health. Intestinal microbiome's role as mediator of inflammation has only recently emerged. Microbiota has been observed to differ in subjects with early rheumatoid arthritis compared to controls, and this finding has commanded this study as a possible autoimmune process. Studies with intestinal microbiota have shown that rheumatoid arthritis is characterized by an expansion and/or decrease of bacterial groups as compared to controls. In this review, we present evidence linking intestinal dysbiosis with the autoimmune mechanisms involved in the development of rheumatoid arthritis.

## 1. Introduction

Rheumatoid arthritis (RA) is a systemic, inflammatory, and chronic disease characterized by a persistent immune response that leads to inflammation and destruction of joints. The etiopathogenic mechanisms involved are complex and include an interaction between the innate and acquired immune response, involving antigen-presenting cells (APCs), autoreactive T cell formation, and production of autoantibodies directed against their own cellular structures, like rheumatoid factor (RF) and anticitrullinated protein antibodies (ACPAs). These antibodies are often present in the blood, long before any sign of joints' inflammation, suggesting that the triggering of autoimmunity might occur at different sites to the joints, for example, the gastrointestinal tract or airway [1]. Epidemiological studies suggest rheumatoid arthritis as the result of complex interactions between genes, environmental and hormonal factors, and the immune system [2, 3].

There is a genetic susceptibility to rheumatoid arthritis by a tendency to familial aggregation, a concordance between monozygotic twins and an association with some histocompatibility antigens [4, 5]. Heritability estimates have suggested a 60–70% genetic risk factors, responsible for developing rheumatoid arthritis [6]. Candidate gene or genome-wide association studies have identified different risk loci associated with rheumatoid arthritis etiology. Currently, in this disease, about 100 described genes are associated with susceptibility, protection, severity, activity, and treatment response [6]. Human leukocyte antigen (HLA) polymorphisms are the most important genetic risk factors. HLA are an important part of the immune system triggering T cells of the immune system to produce antibodies. Associations of RA with HLA-DRB1 alleles have been observed in all racial and ethnic populations [7, 8]. The shared epitope (SE), a 5-amino acid sequence motif in positions 70–74 of the HLA–DR*β* chain, is the most significant genetic risk factor for rheumatoid arthritis [9]. Some SE alleles, such as HLA-DRB1^∗^0401, appear to confer a higher risk than others; moreover, the presence of two SE alleles and in particular HLA-DRB1^∗^0401/^∗^0404 confers a high risk to develop the disease and has also an influence on disease severity [10]. SE alleles are associated with ACPA-positive rheumatoid arthritis, but only relatively weakly with ACPA-negative rheumatoid arthritis [8]. SE alleles might contribute to the genetic predisposition to rheumatoid arthritis causing an immune dysregulation (controlling both specificity and amount of ACPA production) or a premature immunosenescence [10].

In genetically disease-susceptible individuals, subsequent environmental triggers might induce rheumatoid arthritis development. The bacterial and viral components are an attractive source of antigens capable of inducing rheumatoid arthritis and, therefore, have been the most investigated as potential causal agents [3]. However, there is no conclusive evidence to date of a causal relationship of a microorganism with rheumatoid arthritis.

In recent years, characterization and understanding of this gut microbiota has increased and constitutes a wide research field, especially in autoimmune diseases. The gut microbiota is the major source of microbes that may exert beneficial as well as pathogenic effects on human health [11]. Encouraged by studies that show alterations in intestinal microbiota composition in autoimmune diseases, such as rheumatoid arthritis, the interest of studying microorganisms as potential candidates in the development of autoimmunity has been renewed [11–14].

Findings supporting the idea that the onset of autoimmunity may be related to gastrointestinal tract are as follows: (1) microbial composition in subjects with early rheumatoid arthritis differs to controls, with a reduction of certain bacteria belonging to the family *Bifidobacterium* and *Bacteroides* [15, 16], and a marked increase of species belonging to the genus *Prevotella* [17]. (2) In murine models, the parenteral injection of cell wall fragments from various intestinal bacteria is arthritogenic [17] and in this model, arthritis is not developed when bred in germ-free conditions; otherwise, it is presented when intestinal bacterial species are introduced [18]. (3) Diet has been shown to influence inflammatory activity levels [19]. (4) Some drugs used to treat rheumatoid arthritis have antimicrobial effects (chloroquine, sulfasalazine, minocycline, and roxithromycin) [20–23]. (5) Altered microbiome was partially restored to normality in patients showing clinical improvement after prescribing disease-modifying antirheumatic drugs [5, 18]. So, differences in composition of intestinal microbiota and in the immune system function could determine which patients develop the disease.

A great effort is currently being made to study subjects with rheumatoid arthritis in preclinical phase. Awareness of the mechanisms that initiate the autoimmune process, as well as the ones involved in the transition from pre- to clinical phase, might guide to intervention strategies which allow its prevention or treatment in very early stages of the disease. Some publications suggest that rheumatoid arthritis early treatment leads to better long-term outcomes and perhaps to increased rates of drug-free remission [24]. Prevention concept is an emerging research field in rheumatology area, in which modifications of the microbiota could be a new way of modulating the disease.

## 2. Immunopathogenesis of Rheumatoid Arthritis

Understanding the complex molecular processes that play a role in the pathogenesis of rheumatoid arthritis is still a challenge. Susceptible individuals under genetic and environmental factors with loss of immunological tolerance to self-antigens trigger the autoimmune phenomena and the autoantibody formation [25]. This lack of immunological tolerance represents the first step towards autoimmunity. Immune system dysregulation is characterized by the presence of autoantibodies and autoreactive T cells. The inappropriate generation of autoreactive B cells is the most obvious alteration of the immune system in these patients; they are detected long before the disease appears. The most important are RF and ACPAs that recognize different proteins in the citrullinated form [26]. Together with increased autoantibody production, proinflammatory cytokines' level is elevated in the joint synovium of patients with rheumatoid arthritis. The joints of patients with RA are complicated tissues where innate and adaptive immune cells along with joint resident cells, like synoviocytes and chondrocytes, are involved [27].

Multiple cell types have been identified to contribute to the pathogenic context in rheumatoid arthritis. In rheumatoid synovium, dendritic cells are found mainly in lymphocytic aggregates and peripheral vessels, suggesting that they come from peripheral blood. MHC alleles are expressed by APCs that processed extracellular peptides to CD4^+^ T cells, driving the secretion of proinflammatory cytokines that stimulate B cells to produce antibodies [25]. Patients with the disease present a defective function of circulating regulatory T cells (Treg) and an increase in T helper 17 (Th17) cells in plasma and synovial fluid [28]. Macrophage-derived and dendritic cell-derived transforming growth factor *β* and interleukin-1*β*, 6, 21, and 23 provide a milieu that supports Th17 differentiation and suppresses differentiation of regulatory T cells, thus shifting T cell homeostasis towards inflammation [29].

Posttranslational modifications (PTMs) are critical for the function and antigenicity of proteins. The three PTMs primarily involved in rheumatoid arthritis are glycosylation, carbamylation, and citrullination [25]. Citrullination results from the conversion of arginine onto citrulline by the enzyme peptidyl arginine deiminases (PAD), and this is the major crucial posttranscriptional modification associated to self-antigen recognition in rheumatoid arthritis [25]. Citrulline may alter protein structure and generate new epitopes associated with the production of ACPAs. ACPAs present in rheumatoid arthritis patients show differing fine specificities and cross-reactivity degrees with various citrullinated and/or posttranslationally modified peptides/proteins, including fibrinogen, fibronectin, *α*-enolase, collagen type II, and histones [30].

Table 1 summarizes the relevant aspects in the evolution of this disease. Preclinical rheumatoid arthritis comprises the period in which the autoimmunity is detectable until the beginning of the inflammation and/or injury of clinically apparent tissue, genetic and environmental risk factors interact, probably sequentially, to initiate and propagate the development of autoimmunity, finally resulting in detectable tissue inflammation and lesion [24, 31]. If any other risk factor is involved in the onset and/or spread of the disease, it is still unknown.

ACPAs' response might be important to transition from preclinical phase to clinical expression of rheumatoid arthritis. ACPAs' repertoire analysis prior to diagnosis in patients with rheumatoid arthritis revealed that this immune response starts in a very restricted manner and expands to several months or even years (epitope spreading, from one initially recognized epitope towards reactivity to many different epitopes) before the diagnosis of rheumatoid arthritis [10, 24, 32, 33]. Epitope spreading towards more citrullinated ones is compatible with the possibility that a single antigen (but not always the same) is responsible for starting up the immune response [16, 32]. Sokolove et al. [33] reported that earlier identified autoantibodies were targeted against various ligands of the innate immune response including citrullinated histones, fibrinogen, and biglycan. Over time, the ACPAs' titers and epitope diversity of ACPAs increase, especially before arthritis onset. ACPAs could be isotypes IgG, IgA, or IgM with an altered glycosylation status that confers enhanced Fc-receptor and citrullinated antigen binding [34]. ACPAs themselves can be pathogenic by activating either macrophages or osteoclasts via immune complex formation and Fc-receptor engagement or probably by binding membrane citrullinated vimentin, thus promoting bone loss [34].

Since the original description of antibodies as citrullinated antigens in a subpopulation of rheumatoid arthritis patients, it has become clear that citrullinated epitopes of a large number of autoantigens as well as antigens derived from microorganisms can be recognized by highly specific antibodies for rheumatoid arthritis [30]. Alterations at specific mucosal sites suggest that microbial factors might affect mucosal immune response, also playing an important role in early pathogenesis of rheumatoid arthritis [35]. Alterations in compositional diversity and abundance levels of microbiota, that is, dysbiosis, can trigger several types of autoimmune and inflammatory diseases through the imbalance of T cell subpopulations, such as Th1, Th2, Th17, and Treg cells [27].

Dysbiosis in one or more mucosal sites leads to immune alterations and breaks in self-tolerance to citrullinated autoantigens [35]. Mucosal body surfaces such as respiratory and gastrointestinal tract carry out complex tasks as they must (1) remain tolerant against innocuous environmental, nutritional, and microbial antigens to ensure organ function and (2) set efficient immune responses against invading pathogens [36]. Lung and gut tissues contain immunological cells able to initiate an immune response; an attractive possibility is that the triggering of T cell-mediated immunity to citrullinated autoantigens can occur in the mucosae after presentation of neoantigens by APCs [1, 31]. In mucosal site, possible roles for citrullinated microbial antigens and molecular mimicry, Toll-like receptor (TLR) signals, and other innate immune activators and danger signals might exist [35]. Mucosa-associated bacterial flora and smoking or environmental particles (silica dust) act on immune cells (neutrophils, dendritic cells, and macrophages) as pathogen-associated molecular patterns and damage them, leading to inflammation onset, circulating cytokines, and chemokine increase along with autoantigen production. The citrullinated antigens are processed and presented by the APCs to the T cells, which are activated and in turn activate the B cell, leading to the production of autoantibodies [31]. Autoantigens in rheumatoid arthritis are not tissue- and organ-specific but comprise a large collection of posttranslational modified proteins [31]. Smoking and other stimuli might initiate citrullination by PAD activation, formation of lymphoid structures that could enhance antigen presentation, and T and B cell production [31]. Potential mechanisms by which cigarette smoke (CS) promotes rheumatoid arthritis include release of intracellular proteins from reactive oxidative substances activated or injured cells, augmentation of autoreactive B cell function, and alteration of (a) many cell signaling pathways involved in cellular activation, (b) cigarette smoke-impaired antigen-presenting cells, (c) regulatory T cell functions, and (d) T cell activation by antigens found in cigarette smoke [37].

## 3. Microbiota and the Immune System

Microbial exposures in gastrointestinal and respiratory tracts are key determinants of the overall immune tone at these mucosal barriers and represent a leading target for future intervention strategies [36]. Gut is an entryway for various environmental antigens in the form of food or infectious agents. Intestinal microbiota is a factor influencing metabolic homeostasis and the immune system [5]; it is a site of remarkable interaction between microorganisms and human body. Microorganisms establish a symbiotic relationship with epithelial and lymphoid tissue [12, 25]. Intestinal bacteria synthesize and change a variety of compounds that affect physiology and immunity. However, not all host-microbiota interactions promote health, particularly species of resident bacteria seem to activate the immune system resulting in inflammatory diseases [38, 39]. A diverse and balanced microbiota is necessary to develop an appropriate immune response [40].

Benefits provided by gut microbiota to the host rely on intricate interactions with host cells [41]. Studies using germ-free and gnotobiotic animals, colonized with defined bacteria, provided direct evidence about microbiota's crucial role in development and maintenance of the host immune system [41] and preservation of its functions such as maturation of intestinal lymphoid tissue, secretion of immunoglobulin A, and the production of important antimicrobial peptides [42]. In axenic mice, scarce growth of lymphoid tissue and alterations in T cells and subpopulations of B lymphocyte development were observed; in some cases, these mice did not develop diseases presented in ordinary subjects, probably due to defects in the adaptive immune system in the absence of the microbiota, rather than to the absence of microorganisms per se. Conserved molecular patterns, either expressed on the surface of symbiotic bacteria or secreted in the intestine, may interact with pattern recognition receptors (PRRs), which are expressed on or within epithelial and lymphoid cells to initiate transduction and transcription of signals from a set of molecules that mediate host defense or metabolic activities within the gut [40].

Intestinal commensal microbiota have been shown to modulate T cell and Treg responses that are required for effective host defense against pathogens while circumventing autoimmune responses and other immunopathologic consequences [43]. As the first line defense of host against pathogens, innate immune responses rely on a family of receptors known as PRRs including TLRs and nucleotide-binding oligomerization domain-like receptors (NLRs). TLRs are key innate immune receptors to perceive pathogen-associated molecular patterns (PAMPs), which are specific pathogenic “molecular signature.” Subsequent to sensing microbial PAMPs, TLRs enable the initiation of inflammatory responses and eventually eliminate the pathogenic invaders [43]. Components of gram-positive and gram-negative bacteria interact with TLRs to mediate both innate and adaptive immunity, as well as other cellular functions of the mucosal barrier [40]. Epithelial cells have TLRs on their cell membrane, which allow the recognition of PAMPs and the activation of MyD88 coupling protein-mediated signaling that ends with the induction of an inflammatory response and the production of proinflammatory cytokines such as tumor necrosis factor-alpha (TNF-*α*), interleukin-6, or interleukin-1*β*. The innate immune response cells of the lamina propria constantly examine the contents of the intestinal lumen for foreign antigens and constitute another defense mechanism [28]. Because commensal bacteria differ in their ability to stimulate receptors for innate immunity (TLRs and NLRs), the pattern of released chemical mediators varies significantly by determining proinflammatory or anti-inflammatory responses. Lipopolysaccharide of gram-negative bacteria binds to TLR-4, whereas peptidoglycan and other components of the cell wall of gram-positive bacteria signal via the TLR-2 pathway generating an immune response [11]. Gram-positive anaerobic bacteria contain a greater amount of polysaccharides and peptidoglycans that may act as antigenic stimuli.

After interaction with an antigen, dendritic cells play an important role in the differentiation of immature CD4^+^ lymphocytes into Th1, Th17, or Th2 cells. The differentiation of the T helper cells seems to be deeply influenced by the intestinal microbiota [11, 16, 42]. Dendritic cells act as APCs by displaying charged peptides in their MHC class II molecules. Presentation of these molecules to B cells or to T cell receptors sensitizes these cells to initiate an adaptive immune response [28]. In rheumatoid arthritis, dendritic cells could participate in maintenance of inflammatory process by regulating antigenic presentation, and the presentation of the antigen or the arthritogenic antigens would then be abnormally prolonged, which might favor the perpetuation of inflammation. Macrophages and dendritic cells continuously detect intestinal lumen antigens and evaluate the presence of deleterious antigens. Antigens are presented by MHC II molecules and interact with B cells or T cell receptors to induce adaptive immune responses. Depending on the microbial antigen, a specific cytosine medium is then generated to influence a specific type of T helper cell differentiation. While type 1 T helper (Th1) cells develop in response to intracellular pathogens and produce interferon, both type 2 T helper (Th2) cells and Th17 cells are stimulated by extracellular microorganisms. Th17 cells contribute to the defense against extracellular pathogens by the production of IL-17 and IL-22, which induce the change of immunoglobulin class in B lymphocytes and the production of Reg3g by epithelial cells, respectively [14]. The immune response generated by effector T cells is regulated by the subpopulation known as regulatory Treg cells. Intestinal Treg cells play an important role in maintaining immune tolerance to dietary antigens and gut microbiota [44]. Treg cells retain tolerance to self-antigens and eliminate autoimmunity. CD4^+^CD25^+^ Treg cells are suppressive cells, which express the transcription factor Foxp3, and are indispensable for maintenance of immune self-tolerance and homeostasis by suppressing aberrant or excessive immune response. *Lactobacillus* and *Bifidobacterium infantis* exert an anti-inflammatory effect through the induction of CD4^+^CD25^+^FoxP3^+^ Treg cells [11]. *Bacteroides fragilis* polysaccharide A acts as an immunomodulator and stimulates CD4^+^ Treg cells through an interleukin-2-dependent mechanism to produce IL-10 [11].

Taking into account the fact that dendritic cells are fundamental in generating immune response, it has been hypothesized that commensal bacteria influence the function and differentiation of dendritic cells, thus modulating immune response [11, 23]. Thus, intestinal dysbiosis can induce arthritis by influencing the differentiation of T cell subgroups. It also influences the expression degree of Toll-like receptors of antigen-presenting cells and may contribute to an unbalance in the Th17/Treg cell ratio. With the development of Th17 cells, activating local inflammatory cascades with tissue damage and in predisposed individuals, this local immune response can lead to systemic autoimmunity with self-reactive Th17 cells.

The microbiota are the most prominent influence from the environment on the differentiation of Th17 cells. Recently, much attention has been paid to segmented filamentous bacteria (SFB) because of its ability to induce the production and activation of Th17 cells in the intestine, with the secretion of interleukin-17 [23, 27]. SFB comprise a group of Clostridia-related gram-positive bacteria that adhere closely to Peyer's plaques in the small intestine and can stimulate the immune response by inducing IgA secretion and activating B cells. These bacteria are necessary for the development of autoimmunity in the murine K/BxN arthritis model [45], and the use of antibiotics prevents the advance of arthritis [13, 30, 31]. Studies in murine models revealed that induction of T_FH_ and Th17 cells precedes the onset of arthritis, indicating a role for both types of cells. Monocolonization with SFB enhances the production of autoantibodies and accelerates the progression of disease through the generation of Th17 cells although a microbiota-induced T_FH_ cell-dependent process can also precipitate disease [44]. Teng et al. [46] demonstrated that SFB trigger autoimmune arthritis by inducing differentiation and migration of gut T follicular helper cells (T_FH_) to systemic lymphoid sites, leading to increased autoantibody production and arthritis exacerbation. In contrast, Block et al. [45, 47] confirmed a role for gut microbiota in the differentiation of T_FH_ cells and germinal center formation. Depletion of gut microbiota in mice by antibiotics reduced the number of T_FH_ cells and antibody production levels. They concluded that intestinal microbiota regulates the development of arthritis through T_FH_ independently of Th17 cells.

## 4. Dysbiosis in Rheumatoid Arthritis

Importance of gut microbiome in autoimmunity related to rheumatoid arthritis has been implicated in both mouse models and human disease. Alterations of microbiota are related to risk and severity of the disease. Three sites have been associated particularly with it, mainly the lungs, oral mucosa, and gastrointestinal tract. However, the site(s) of initial immune response triggering remains to be verified. Whereas precise mechanisms that enhance risk are not fully understood for each, it is likely that local tissue stress leads to posttranslational modification of peptides with subsequent antibody formation serving as a common mechanism [48].

Airway abnormalities and lung tissue citrullination are found in both rheumatoid arthritis patients and individuals at risk. This suggests the lung as a possible site of autoimmunity generation [49]. Evidence of an early role of the adaptive immunity and immune activation in the lungs of these patients comes from a proteomic study where two shared citrullinated vimentin peptides have been described in bronchial tissue form early rheumatoid arthritis patients and synovial tissue from patients with established disease, offering some clues on an immune process initiated in the lungs [25]. The pulmonary mucosa as an autoimmune process origin is based on the following observations: ACPAs are presented in the sputum of ACPA-positive patients without arthritis; there are microscopic and macroscopic changes in the lungs with early rheumatoid arthritis and untreated ACPA-positive patients; pulmonary alterations have been demonstrated in high-resolution computed tomography in subjects without the disease but with ACPA-positive; pulmonary biopsy samples from patients with ACPA positive with established AR suggest that ACPAs are produced locally. However, the precise molecular mechanisms that could be responsible for the triggering of immunity in the lung mucosa are relatively unexplored. It is known that lung exposure to harmful agents, including smoke, may induce increased expression and activation of PAD [1]. It has been hypothesized that the citrullinated proteins may become an autoantigen and thereby trigger an immune system response in people with genetic predisposition for rheumatoid arthritis.

Smoking and periodontitis promote protein citrullination and ACPA production [50]. Attention in the gums is based on periodontal disease, more frequent in subjects with rheumatoid arthritis or correlated with its activity. It has been speculated that periodontal pathogens drive systemic inflammation or disseminate to affected tissue. Indeed, increased specific IgG to periodontal pathogens, including *Prevotella intermedia* and *Porphyromonas gingivalis*, has been reported in RA [51]. The presence of *P. intermedia* and *P. gingivalis* in the subgingival dental plaque, as well as synovial fluid, supports a role of microbiota in initiating or maintaining chronic inflammation [52]. Cross-sectional studies have revealed correlations between RA and higher titers of serum antibodies against proposed periodontal pathogens such as *P. gingivalis*, *Prevotella melaninogenica*, and *Tannerella forsythia* [53]. Oral inoculation with *P. gingivalis* and *Prevotella nigrescens* aggravated the severity of arthritis in an experimental mouse model by directing the immune pathway towards production of IL-17 and generation of a Th17 response [54]. The effects of the two bacteria diverged in that *P. nigrescens*, in contrast to *P. gingivalis*, suppressed the joint-protective type 2 cytokines, including IL-4. *P. gingivalis* expresses an enzyme with amino deiminase activity that converts C-terminal arginine to citrulline, similar to the one involved in the etiology of rheumatoid arthritis. Citrullination of bacterial and human proteins by PAD can expose hidden epitopes leading to tolerance loss in genetically susceptible individuals. It is believed that the resulting immune response together with endogenous citrullination produces ACPAs [4]. The fact that smoking is strongly linked to the presence of periodontitis and *P. gingivalis* infection provides circumstantial evidence in favor of this hypothesis. However, recent epidemiological data have not demonstrated a clear relationship between periodontitis and rheumatoid arthritis [1].

Recently, gut microbiota has been proposed as an indispensable environmental factor in the progression of rheumatoid arthritis [5, 18, 27, 55–57]. Bennike et al. [43] identified 21 citrullinated peptides in the colonic tissue from both rheumatoid arthritis patients and controls, which have been previously found in lung tissue and synovial fluid from RA patients. Three citrullinated proteins (citrullinated vimentin, fibrinogen-alpha, and actin) are known targets for ACPAs, supporting that colon mucosa could be a potential break site for immune tolerance towards citrullinated epitopes. Citrullinated vimentin was found with increased abundance in the colonic tissue of these patients compared to the controls, which could indicate that initial rheumatoid arthritis triggering is not limited just to a specific location in the body but can take place in many other locations [43]. Therefore, this study supports the hypothesis that colon mucosa could serve as a break site for immune tolerance to citrullinated proteins, triggering ACPA production in patients with impaired immune system.

## 5. Gut Microbiota in Rheumatoid Arthritis

Potential role of intestinal microbiota in etiopathogenesis of rheumatoid arthritis is supported by studies in animal models, by research on intestinal microbioma, and indirectly, by the effect of diet and probiotics in the degree of inflammatory activity. Pathophysiologic mechanisms by which gut microbiota is associated with arthritis is probably multifactorial; proposed mechanisms include activation of antigen-presenting cells through an effect on TLRs or NLRs, ability to produce citrullinization of peptides by enzymatic action, antigenic mimicry, alterations in permeability of intestinal mucosal, control of host immune system (triggering T cell differentiation), and increase of T helper type 17-mediated mucosal inflammation.

Despite the existence of animal models, it is well known that there is no animal model that represents rheumatoid arthritis entirety [58]. However, murine arthritis models have shown to induce erosive polyarthritis by intraperitoneal injection of cell wall fragments of *Streptococcus pyogenes*, *Lactobacillus casei*, and *Eubacterium aerofaciens* [59–61]. The arthritogenicity of bacterial structures depends on bacterial species, and remarkably, even bacteria from the normal intestinal microbiota cause experimental arthritis in animals [17]. Gnotobiotic mice do not develop arthritis, and introduction of SFB is enough to reintegrate Th17 cells from the lamina propria, a greater production of autoantibodies and a rapid development of destructive arthritis [62]. Antibiotic treatment prevents and suppresses a phenotype similar to rheumatoid arthritis in several murine models [4, 18, 28], and in genetically susceptible mice, dysbiosis increases sensitivity to arthritis through activation of autoreactive T cells in the gut [63].

Intestinal microbiota's role in pathogenesis of arthritis was demonstrated by the induction/exacerbation of arthritis in experimental murine models [55, 64–66]. Studies with gnotobiotic mice have shown that disruptions in the intestinal microbiota could induce production of proinflammatory cytokines, interleukin-17, and increased levels of Th17 cells, even in extraintestinal tissues [11]. Th17 cells then migrate into the peripheral lymphoid tissue and secrete IL-17, which in turn, acts directly on B cells and induces systemic B cell differentiation and antibody production [67]. This ultimately can lead to development of autoimmune disease via molecular pattern recognition from gut microbiota [67]. The IL-1 receptor antagonist (IL-1Ra) knockout mice, which spontaneously develop autoimmune T cell-mediated arthritis, do not develop disease when raised in a germ-free environment. However, colonization with commuter *Lactobacillus bifidus* produces a rapid onset of the disease, severity, and incidence comparable to the arthritis observed in mice. *L. bifidus* triggers arthritis in this model by promoting an imbalance in Treg–Th17 cell homeostasis and mediated through Toll-like receptor signaling (TLR2-TLR4) [28, 65]. Liu et al. [64] found that the genus *Lactobacillus* was significantly more abundant in collagen-induced arthritis- (CIA-) susceptible mice prior to arthritis onset than in CIA-resistant mice. Notably, germ-free mice conventionalized with the microbiota from CIA-susceptible mice showed a higher frequency of arthritis induction than those conventionalized with the microbiota from CIA-resistant mice. Monocolonization of germ-free K/BxN mice with SFB was sufficient to drive production of autoantibodies and pathogenic Th17 cells as well as to trigger arthritis [66]. Systemic deficiencies of germ-free animals reflect a loss of Th17 cells from the intestinal lamina propria. Introduction of a single species of resident intestinal filamentous bacteria restored the Th17 cell compartment into the lamina propria; autoantibodies production and arthritis occurred rapidly. Thus, a single commensal microbe, through its ability to promote a specific subpopulation of T helper cells, can lead to an autoimmune disease [66]. These results provide the evidence that commensal bacteria can drive autoimmune arthritis by inducing a Th17 response in the intestine. Therefore, composition of gut microbiota plays a pivotal role in the balance between inflammatory Th cells and suppressive Treg cells to maintain immune tolerance under healthy conditions [27].

Interaction between host genetic factors such as MHC and intestinal microbiota and its impact on development of rheumatoid arthritis is difficult to study in humans because of the high variability of genetic factors and diet [11]. Research conducted by Gomez et al. [68] provided the first demonstration that HLA genes and intestinal environment interact to affect the susceptibility of arthritis. This study showed differences in fecal microbiome of rheumatoid arthritis-susceptible HLA-DRB1^∗^0401 transgenic mice compared to DRB1^∗^0402 mice resistant to development of rheumatoid arthritis. Specifically, DRB1^∗^0401 female mice had significantly different microbial to DRB1^∗^0402 females, and this resulted in an increase in intestinal permeability and transcripts of Th17 type cytokines in DRB1^∗^0401 mice. The analysis showed that the intestinal flora of arthritis-susceptible mice had a greater amount of *Clostridium* sp. bacteria, while those of DRB1^∗^0402 resistant to arthritis were enriched by the families *Porphyromonadaceae* and *Bifidobacteriaceae* [68]. The latter organism has been associated with the anti-inflammatory response in the intestinal mucosal immune systems through the suppression of T cell proliferation and the production of proinflammatory cytokines and by inhibition of nuclear factor kB. The results show a difference in intestinal microbial composition between the two strains, suggesting that MHC genes may be directly or indirectly involved in determining the intestinal microbial composition and that interactions between bowel commensals [13, 18]. In addition, they demonstrated that dysbiosis is not enough since it requires a genetic susceptibility of the host, due to an induction inability in inflammatory response in wild animals, even with proarthritogenic intestinal flora [13, 28].

Several studies attempting to link gut mucosal and joint inflammation have been followed during the past decades. Eerola et al. [69] reported that fecal profile of bacterial cell fatty acids was significantly different in subjects with rheumatoid arthritis, mainly by anaerobic bacteria compared to controls. They support the idea that rheumatoid arthritis represents a state of chronic inflammation that could be motivated or aggravated by pathogenic bacteria overgrowth or by a lack of common immunomodulating bacteria [5, 16, 18, 57, 70]. Vaahtovuo et al. [16] also described differences in fecal diversity in individuals with rheumatoid arthritis compared to those with fibromyalgia, characterized by lower bundi-bacteria, *Bacteroides-Porphyromonas-Prevotella*, subgroup *Bacteroides fragilis*, and the group *Eubacterium rectale-Clostridium coccoides* in subjects with rheumatoid arthritis [6]. On the other hand, Newkirk et al. [71] identified differences in the types of *E. coli* pathogen colonization among subjects with rheumatoid arthritis, RF-positive patients were more commonly colonized with *E. coli*, phylogenetic group D, whereas RF-negative patients were more commonly colonized with *E. coli*, phylogenetic group B2, and these individuals also had lower joint scores and inflammatory markers yet higher IgA anti-*E. coli* antibody responses.

The observation that gut microbiota differs in subjects with early rheumatoid arthritis compared to controls has renewed the interest in studying intestinal microbiota as a possible site of origin of the autoimmune proces; studies that evaluate the intestinal microbiota show that rheumatoid arthritis is characterized by an expansion and/or decrease of bacterial groups as compared to controls [5, 18, 55–57]. High-throughput sequencing of stool samples in 5 studies of RA patients showed gut dysbiosis [5, 18, 55, 57, 63], and 2 study reported overexpansion of *Prevotella* sp. in patients with early RA, particularly *P. copri* (Table 2). Differences between the bacteria reported in the studies may be influenced by the time course of the disease (i.e., early versus established), subjects included in the control groups (healthy or first degree relatives), the treatment received, and the geographical location, since the studies do not show the same pattern.

Toivanen et al. [57] compared fecal microbiota from 25 patients with early RA to the microbiota of patients with noninflammatory pain using oligonucleotide probe against 16S RNA. Patients with early RA had significantly fewer bacteria belonging to the gender *Bacteroides* sp., *Prevotella* sp., and *Porphyromonas* sp. In other study, Scher et al. [18] reported that individuals with early RA were more likely to harbor *Prevotella copri* compared to controls. In addition, they demonstrated that oral administration of *P. copri* increased local inflammatory response in a colitis murine model. Therefore, *P. copri* would alter intestinal permeability. Such increase might lead to bacteria penetration and/or its components throughout the body; this is one of the proposed mechanisms that link dysbiosis with the pathogenesis of arthritis. Interestingly, the relative abundance of *P. copri* showed a negative correlation with the presence of shared epitope, suggesting that the composition of the human intestinal microbiome could also be partially dependent on the host genome and suggesting a dysbiosis before the appearance of the clinical phenotype.

In line with these results, Maeda et al. [63] observed that *P. copri* was in abundance within gut microbiota in Japanese patients with early RA who had not received drug treatment. They identified that *P. copri* per se had a high capacity to induce Th17 cell-related cytokines, such as IL-6 and IL-23. Increased *Prevotella* sp. abundance is associated with augmented T helper type 17-mediated mucosal inflammation, which is in accordance with the marked capacity of *Prevotella* sp. in driving Th17 immune responses in vitro [51]. In other study, Pianta et al. [72] identified that subgroups of rheumatoid arthritis patients have differential IgG or IgA immune reactivity with *P. copri.* In both new onset rheumatoid arthritis patients and chronic ones, a subgroup had IgA antibody responses to either Pc-p27 or the whole organism, which correlated with Th17 cytokine responses and frequent ACPAs. The second subgroup had IgG *P. copri* antibodies, which were associated with *Prevotella* DNA in synovial fluid, *P. copri*-specific Th1 responses, and less frequent ACPAs. Patients with RA had IgA, IgG, or no specific antibodies, indicating that different immune responses to *P. copri* can develop within the individual patient, which in turn may have implications for disease risk and outcomes.

Molecular mimicry, due to its sequence similarities between foreign and self-peptide, is one such mechanism known to result in cross-activation of pathogen-derived autoreactive T or B cells. These T and B cells can cross-react with host epitopes, thus leading to autoimmunity [67]. In a recently work, Pianta et al. [73] described two proteins derived from common types of gut bacteria that could evoke the immune responses in RA patients. *N*-Acetyl-glucosamine-6-sulfatase (GNS) and filamin A (FLNA) were identified as autoantigens that produce responses from both T and B cells, in over 50% of RA patients, but not in healthy controls or patients with other rheumatic diseases. The HLA-DR-presented GNS peptide has marked sequence homology with epitopes from sulfatase proteins of the *Prevotella* sp. and *Parabacteroides* sp., whereas the HLA-DR-presented FLNA peptide has homology with epitopes from proteins of the *Prevotella* sp. and *Butyricimonas* sp., another gut commensal. T cell responses to the corresponding microbial and self-peptides were strongly correlated. These study provides evidence that T cell epitopes of a related order of gut microbes, particularly *Prevotella* sp., may cross-react with self-epitopes of highly expressed proteins in joints, especially in patients with SE alleles.

Moreover, *Prevotella* sp. may not be the only genus participating in inflammatory disease. Zhang et al. [5] used metagenomic shotgun sequencing technology to analyze fecal, dental, and salivary samples from a large cohort of RA patients as well as from healthy controls. Analysis of differentially represented metagenomic linkage groups revealed significant microbiome differences between RA patients and healthy controls, not only in fecal but also in salivary and dental samples. They demonstrated that gram-positive bacteria enriched microbiota versus few gram-negative bacteria and, furthermore, that dysbiosis was associated with inflammation markers and clinical rheumatoid arthritis activity. Besides, they also detected alterations in redox environment and transport and metabolism of iron, sulfur, and zinc in the microbiota of RA patients, indicating that the altered microbiome could play an important role in the pathogenesis of RA. Noteworthy, altered microbiome was partially restored to normal (microbiome of healthy controls) in patients who showed clinical improvement after prescribing disease-modifying antirheumatic drugs. Remarkably, *P. copri* in RA-affected individuals showed a trend of increasing relative abundance in the first year, consistent with its reported expansion in early RA. Many *Prevotella* species were enriched in the saliva of RA subjects compared with controls. One notable exception was *Prevotella intermedia*, which was enriched in the control group. However, in dental plaque samples, there was a very different picture and most *Prevotella* sp. were present at higher proportions in healthy subjects.

Chen et al. [55] reported that bacteria belonging to the phylum *Actinobacteria* play a significant role in the pathogenesis of rheumatoid arthritis, *Collinsella* sp. and *Eggerthella* sp. predicted its presence, and dysbiosis in the intestinal microbiome is partially restored after treatment with disease-modifying antirheumatic drugs, similar to results reported in the previous study. The potential association of *P. copri* as previously reported with new onset untreated RA and DR4 was not observed in this cohort of RA patients (in contrast to previous studies, all the patients in the present study were currently on a treatment regimen). They showed that the decreased gut microbial diversity of RA patients is associated with disease duration after adjusting for various drugs used for treatment. *Collinsella* sp. may contribute to the development of rheumatoid arthritis through molecular mimicry as it presents sequences shared with DRB1^∗^0401. The role of the RA-associated bacteria *Collinsella* sp. was confirmed using a human epithelial cell line and a humanized mouse model of arthritis. *Collinsella* sp. enhanced disease severity in a humanized mouse model. One mechanism by which Collinsella contributes to disease pathogenesis is by increasing gut permeability as observed by the lower expression of tight junction proteins. Additionally, Collinsella influences the epithelial production of IL-17A [55].

Although studies in subjects with rheumatoid arthritis show a correlation of *Prevotella* sp. in its pathogenesis, other studies suggest that it may be considered a beneficial bacterial species rather than pathogenic [74, 75]. Culture collections now include approximately 40 different *Prevotella* species, most of them oral isolates, and three of which are found in the gut (*P. copri* is generally the more abundant). The vastly different genome gene repertoires of strains within and between *Prevotella* sp. and across hosts probably underlie some of the differences observed in responses at the genus level to diet and health conditions across individuals [75]. A recent comprehensive study comparing several bacterial species suggests that membership of a specific phylum does not predict immunological properties, underlining the importance of characterizing properties at species level. Marietta et al. [76] evaluated the ability of 2 species of *Prevotella* sp. for arthritis prevention and treatment in HLA-DQ8 mice. It was shown that the ability to modulate the immune response differed between the *Prevotella* strains. Treatment with *P. histicola* suppressed arthritis development by modulating the immune response (regulation of dendritic cells and generation of Treg cells), resulting in suppression of Th17 responses and reduction of inflammatory cytokines (IL-2, IL-17, and tumor necrosis factor). In contrast, administration of *P. melaninogenica* showed no significant change in cytokine levels, either preventing arthritis development. The ability of *P. histicola* to modulate was validated using a DBA/1 mouse model, demonstrating that mice treated with *P. histicola* developed milder arthritis compared to controls. It is clear that a single strain of *Prevotella* sp. can act in what has been interpreted as a beneficial or detrimental manner, depending on the context. This may explain why *Prevotella* sp. is abundant in healthy microbiota and suggests that only certain strains may exhibit pathogenic properties.

In new onset RA patients, *Prevotella* abundance in the gut was at the expense of *Bacteroides fragilis*, an organism that is important for Treg function [18, 72]. In fact, high levels of *P. copri* and similar species are related to low levels of beneficial microbes, which are believed to suppress the immune system and metabolize vitamins in forms absorbed into the bloodstream [17]. When discussing possible mechanisms by which diet could influence rheumatoid arthritis, effects of intestinal flora should be considered [77–79]. A diet rich in protein and animal fat is associated with the presence of *Bacteroides* sp. while a diet rich in carbohydrates is associated with the presence of *Prevotella* sp. [80]. Studies have shown that Mediterranean or vegan diet reduces inflammatory activity, increases physical function, and improves vitality [19]. However, some other studies find benefits without achieving a significant improvement in composite indices to measure disease activity [81, 82]. Nevertheless, in these studies, it has not been determined whether a rheumatoid arthritis improvement was actually due to a change in intestinal flora composition.

Probiotics are living organisms that can confer a health benefit to the host. Probiotics mainly exert their beneficial effects through the following three methods: antimicrobial effects, enhancement of mucosal barrier integrity, and immune modulation [83]. Studies [84–86] assessing the relationship between probiotic supplementation with the level of RA activity have not shown conclusive results (Table 3). Improvements in probiotic interventions have not been evident enough to demonstrate the effectiveness of treating RA patients, due to the limited studies. Better-designed research needs to be conducted in order to identify the best species that relieve RA and optimize the intake method and doses of the probiotics [83].

Finally, evidence that intestinal microbiota motivates arthritis development comes from murine experimental models. In humans, this association is based in differences between microbiome within comparison groups, which do not necessarily represent its causality. Results in humans suggest dysbiosis as a significative etiologic agent promoting rheumatoid arthritis progression or, even more, that inflammation caused by some microorganisms like *P. copri*, may probably contribute to arthritis maintenance. In order to predict intestinal microbiota's role in pathogenesis of rheumatoid arthritis, an accurate comprehension related to this species potential, its ecology and interaction with other microbes and/or hosts would still be necessary. Furthermore, although gut mucosal got probably the highest potential for host-microbe interaction, all mucosae have resident microbiota and react dynamically to its presence with an immune modulation. Gram-negative bacteria could cause infections at any part of the body; among the most common types are oral, dental, pleuropulmonar, intra-abdominal, genital mucosal, skin, and soft tissues. In other words, it does not mean that all subjects with rheumatoid arthritis must have dysbiosis in a single site.

## 6. Microbiota as a Possible Mechanism for Rheumatoid Arthritis Prevention

Current research projects are focused on prevention with biological drugs that inhibit antibody formation or activate T cells [87]. Recent findings showed intestinal dysbiosis as a major advance in our understanding of rheumatoid arthritis. Nevertheless, there is no study demonstrating the antigen or antigens that trigger the autoimmune process. Logical indications point mucosal dysbiosis as an attractive site for elucidating autoimmunity pathways, particularly the mechanisms that induce loss of immune tolerance and specific mechanisms by which a person evolves from a preclinical to a clinical disease.

Gut microbiota has been shown to play role in rheumatoid arthritis although the mechanism of this association remains obscure. Understanding these mechanisms is crucial for a better treatment efficacy and personalized patient management [67]. Plasticity of microbiome may allow a specific or systematic manipulation of a certain intestinal microbiota associated with host diseases [88], speculating that, in the future, this manipulation could change therapeutic strategies in subjects with rheumatoid arthritis.

## Figures and Tables

**Table 1:** Natural history of rheumatoid arthritis.

Phase of initiation of the disease (interaction between genetic-hormonal-environmental factors)			Preclinical RA	Clinical RA
Genetic and epigenetic factors	Hormonal factors	Environmental factors	*Immunological changes*	*Immunological changes*
Shared epitope, PTPN22, STAT4, CTLA4, TRAF1, PADI4, FCRL3, TNFIP3 DNA methylation Dysregulated histone marks	Relationship man : woman 4 : 1 Arthritis is improved in the pregnancy but relapse in the postpartum	Microbiota oral, pulmonary and intestinal Smoking Silica dust Obesity Diet	Inadequate response to peptides Expansion of autoreactive T cells and B cells Expansion of antibody isotype usage and class switching Changes in soluble cytokine and chemokine networks Altered Th17 cells and Th17/regulatory T cell ratios	Upregulation of signalling molecules Immune-mediated tissue inflammation Alterations of autoantibodies, such as glycosylation Cellular expansion
			*Clinical manifestations*	*Clinical manifestations*
			Presence of autoantibodies (RF, ACPAs) Nonspecific symptoms	Arthritis Bone erosions Systemic symptoms
*Forms of intervention*				
Suspension of smoking Avoid exposure to silica Healthy diet Maintaining an adequate weight Modifications of the microbiota?			In research, the early use of rituximab or abatacept Modifications of the microbiota?	Anti-inflammatory Biological and nonbiological disease-modifying drugs Glucocorticoids

**Table 2:** Summary of studies that have evaluated the influence of gut flora on the etiopathogenesis of RA.

Author (year of publication)	Design	Subjects included	Method employed	Results in the RA group versus the controls
Shinebaum et al. [70]	Case-control	25 patients with RA compared with controls	Estimation of bacterial counts in fecal culture	Significantly higher carriage rate of *Clostridium perfringens* in the RA population than controls (88% versus 48%, *p* < 0.01). Coliform counts also tended to be higher
Eerola et al. [69]	Case-control	74 treatment-naive early RA and 91 non-RA controls	Gas-liquid chromatography of bacterial CFAs	Variation in CFA profile of RA as compared to controls likely caused by anaerobic bacteria
Vaahtovuo et al. [16]	Case-control	50 individuals with RA and 50 individuals with fibromyalgia	Flow cytometry, 16S rRNA hybridization, and DNA-staining	The RA patients had significantly less bifidobacteria and bacteria of the *Bacteroides-Porphyromonas-Prevotella* group, *Bacteroides fragilis* subgroup, and *Eubacterium rectale*-*Clostridium coccoides* group
Toivanen et al. [57]	Case-control	25 treatment-naive individuals with early RA patients and 23 control patients suffering from noninflammatory pain	16S ribosomal DNA	Patients with early RA had significantly less bacteria belonging to the *Bacteroides*, *Prevotella*, and *Porphyromonas* genera than the controls (4.7% versus 9.5%, *p* < 0.01). The number of bacteria belonging to the *Bacteroides-Prevotella-Porphyromonas* group was, on average, in RA patients only half that of the controls
Scher et al. [18]	Cross-sectional	44 treatment-naive individuals with RA, 26 treated RA, 16 patients with psoriatic arthritis, and 28 healthy controls	16S ribosomal DNA	Increases in *Prevotella copri* (75% versus 21.4%) abundance and decrease in *Bacteroides*
Liu et al. [56]	Case-control	15 individuals with early RA and 15 healthy controls	Quantitative real-time PCR	Fecal microbiota of RA patients contained significantly more *Lactobacillus* (10.62 ± 1.72 copies/g) than the control group (8.93 ± 1.60 copies/g)
Zhang et al. [5]	Cohort	77 treatment-naive individuals with RA and 80 unrelated healthy controls; 17 treatment-naive individuals with RA paired with 17 healthy relatives; and 21 samples from DMARD-treatedindividuals with RA	Metagenomic shotgun sequencing and a metagenome-wideassociation study	The RA gut was enriched in gram-positive bacteria and depleted of gram-negative bacteria, including some *Proteobacteria* and *gram-negative Firmicutes* of the Veillonellaceae family. The RA-enriched MLGs formed a large cluster including *Clostridium asparagiforme*, *Gordonibacter pamelaeae*, *Eggerthella lenta*, and *Lachnospiraceae bacterium.* There was a trend towards increased abundance of *P. copri* as a function of RA duration in the first year
Maeda et al. [63]	Cross-sectional	25 treatment-naive individuals with early RA patients and 23 healthy controls	16S rRNA-based deep sequencing	A subpopulation of early RA patients harbored intestinal microbiota dominated by *Prevotella copri*
Chen et al. [55]	Case-control	40 Subjects with RA with treatment	16S ribosomal DNA	Increased number of reads from the phylum Actinobacteria in the RA group (0.45 versus 0.04%)
		32 controls (15 relatives of 1 degree with AR and 17 healthy subjects)		Decrease in Faecalibacterium and expansion of Collinsella aerofaciens and Eggerthella lenta

MLGs: metagenomic linkage groups.

**Table 3:** Summary of studies evaluating the effect of probiotics on the level of rheumatoid arthritis activity.

Author (year of publication)	Design	Population and intervention	Outcomes
Hatakka et al. [89]	Clinical trial	*Lactobacillus* group (*n* = 8). Placebo group (*n* = 13)	There were no statistically significant differences in: CRP, ESR, IL-6, IL-10, IL-12, and HAQ
Pineda et al. [86]	Clinical trial	15 subjects Lactobacillus rhamnosus GR-1 and *Lactobacillus reuteri* RC-14 administered for 3 months versus 14 subjects in the control group	Probiotics did not statistically improve the percentage of ACR 20 response (20% versus 7%, *p* = 0.33)
Alipour et al. [84]	Clinical trial	22 subjects with *Lactobacillus casei* versus 24 subjects with placebo	Statistically significant decrease in CRP level, count of inflamed and painful joints and in DAS-28
Vaghef-Mehrabany et al. [85]	Clinical trial	22 subjects with *Lactobacillus casei* versus 24 subjects with placebo (maltodextrin)	Statistically significant decrease in level of TNF-*α*, IL-6, and IL-12 count of inflamed and painful joints and in DAS-28

CRP: C-reactive protein; DAS28: disease activity score-28; IL: interleukin; ESR: erythrocyte sedimentation rate; ACR: American College of Rheumatology.
